# Cytogenetic as an Important Tool for Diagnosis and Prognosis for Patients with Hypocellular Primary Myelodysplastic Syndrome

**DOI:** 10.1155/2014/542395

**Published:** 2014-08-11

**Authors:** Daiane Corrêa de Souza, Cecília de Souza Fernandez, Adriana Camargo, Alexandre Gustavo Apa, Elaine Sobral da Costa, Luis Fernando Bouzas, Eliana Abdelhay, Teresa de Souza Fernandez

**Affiliations:** ^1^Bone Marrow Transplantation Center, National Cancer Institute (INCA), 20230-130 Rio de Janeiro, RJ, Brazil; ^2^Mathematical and Statistical Institute of Federal Fluminense University (UFF), 24020-140 Niterói, RJ, Brazil; ^3^Arthur Siqueira Cavalcanti Hematology Institute (HEMORIO), 20211-030 Rio de Janeiro, RJ, Brazil; ^4^Hematology Service, National Cancer Institute (INCA), 20230-130 Rio de Janeiro, RJ, Brazil; ^5^Pediatric and Puericulture Martagão Gesteira Institute, Federal University of Rio de Janeiro, 21941-590 RJ, Brazil

## Abstract

We analyzed cytogenetically 105 patients with hypocellular primary MDS and their clinical implications. The main chromosomal abnormalities found were del(5q)/−5, del(6q)/+6, del(7q)/−7, del(11q), and del(17p). Pediatric patients had a higher frequency of abnormal karyotypes compared with adult patients (*P* < 0,05). From our patients, 18% showed evolution of the disease. The chromosomal abnormalities presented in the diagnosis of patients who evolved to AML included numerical (−7, +8) and structural del(6q), del(7q), i(7q), t(7;9), i(9q), and del(11q) abnormalities and complex karyotypes. Although the frequency of evolution from hypocellular MDS to AML is low, our results suggest that some chromosomal alterations may play a critical role during this process. We applied the IPSS in our patients because this score system has been proved to be useful for predicting evolution of disease. When we considered the patients according to group 1 (intermediate-1) and group 2 (intermediate-2 and high risk), we showed that group 2 had a high association with respect to the frequency of abnormal karyotypes (*P* < 0,0001), evolution of disease (*P* < 0,0001), and mortality (*P* < 0,001). In fact, the cytogenetic analysis for patients with hypocellular primary MDS is an important tool for diagnosis, prognosis, in clinical decision-making and in follow-up.

## 1. Introduction

Myelodysplastic syndrome (MDS) comprises a heterogeneous group of clonal bone marrow disorders characterized by various degrees of pancytopenia and morphological and functional abnormalities of hematopoietic cells and an increased risk of transformation into acute myeloid leukemia (AML) [1]. MDS is known as a disease of adults, particularly the elderly. Pediatric MDS is an uncommon disorder, accounting for less than 5% of hematopoietic malignancies [2, 3]. The bone marrow in primary MDS patients is usually hypercellular or normocellular; however between 10% and 20% of patients can present hypocellular bone marrow [4–6].

The remarkable progress in understanding the leukemogenesis was sustained by methodological developments in the cytogenetic field. The cytogenetic abnormalities have provided molecular basis for the discovery of the genes involved into the leukemogenesis mechanisms. In several studies, the cytogenetic turned out to be one of the most important prognostic parameters and it was incorporated into statistical models aiming for a better prediction of the individual prognosis [7]. In primary MDS, the discovery of nonrandom chromosomal abnormalities confirmed the clonality, providing a way to identify the malignant clone and point out some oncogenes and tumor suppressor genes, possibly involved in the development and in the leukemic transformation. The cytogenetic evaluation of a bone marrow sample from patients with MDS has become an integral part of clinical care [8, 9]. The clonal cytogenetic alterations can be detected in 30–50% of adult patients with primary MDS. In pediatric patients, this incidence is 50–70% of the cases. The most frequent chromosomal abnormalities in MDS are del(5q), del(7q)/−7, +8, del(11q), del(12p), del(17p), del(20q), and loss of Y chromosome [7, 10].

Reviewing the literature, we can notice that there are few studies in hypocellular primary MDS relating the frequency of abnormal karyotypes, their correlation with the subtypes and leukemic transformation [6, 11, 12]. Some authors suggested that the frequency of abnormal karyotypes in hypocellular primary MDS is less than in normo-/hypercellular MDS [11, 12]. However, Yue and colleagues suggested there is no difference in the frequency of abnormal karyotypes between hypocellular and normo-/hypercellular MDS [6]. As we can notice, these studies showed controversies about the frequency of abnormal karyotypes in hypocellular MDS. Several studies have demonstrated the prognosis value of cytogenetic analysis in MDS [10, 11, 13, 14]. So, the karyotype was incorporated in prognostic scores, allowing risk group stratification and helping to choose the therapy like the International Scoring System for Evaluating Prognosis (IPSS). The IPSS divides MDS patients in four risk groups: low, intermediate-1, intermediate-2, and high risk. The parameters used to stratify the patients according to risk groups are percentage of bone marrow blasts, cytogenetic pattern, and number of cytopenias [15]. This score system is the most used in MDS, but it is not well known if it can be used in patients with hypocellular MDS. The hypocellularity in MDS is considered an independent factor which predicts a favorable outcome. However, about 10–16% of the cases showed evolution to AML [6, 12]. In spite of these studies, the chromosomal abnormalities involved for the leukemic transformation in the cases of hypocellular primary MDS are still unknown. In this study, we analyzed the chromosomal pattern of hypocellular primary MDS in pediatric and adult patients, the frequency of chromosomal alterations, its correlation with the different subtypes and with the disease evolution. We analyzed the chromosomal abnormalities during leukemic transformation and we suggested the involvement of genes associated with these cytogenetic abnormalities. We also discussed the application of the IPSS for patients with hypocellular primary MDS.

## 2. Material and Methods

### 2.1. Patients

Bone marrow cells were obtained from a total of 105 patients with hypocellular primary MDS. These patients were studied between 1991 and 2013. Chromosomal and clinical studies were carried out in all cases. The patients were diagnosed at the Hematology/Oncology Centers of some hospitals in Rio de Janeiro, Brazil: Bone Marrow Transplantation Center (CEMO-INCA), Hematology Service (INCA), Arthur Siqueira Cavalcanti Hematology Institute (HEMORIO), and Martagão Gesteira Pediatric and Puericulture Institute (IPPMG). From 105 patients, there were 56 males and 49 female, and the mean age was 31 years, with a range from 1 to 84 years. None of these patients were previously treated for a malignancy. Diagnosis was based on morphological, cytochemical studies and immunophenotypic and cytogenetic analyses. The adult patients were classified according to FAB criteria [16] and the pediatric patients were classified according to Hasle and colleagues [17]. This study was approved by Ethics Committee of National Cancer Institute and all procedures performed followed the bioethics standard, according to resolution 196/96 of Health National Committee.

### 2.2. Conventional and Molecular Cytogenetic Analysis

Karyotypes of bone marrow cells were obtained from cultures in RPMI 1640, with 20% fetal calf serum (GIBCO) at 37°C for 24 hours. Cell cultures were pulsed with colcemid to a final concentration of 0.05 *µ*g/mL for the final hour of incubation. Cells were subsequently harvested by standard procedures (hypotonic shock: 0,075 M) and fixed in methanol: acetic acid (3 : 1). GTG banding was performed. Chromosomes were identified and arranged according to the International System for Cytogenetic Nomenclature, 2013 [18].

Fluorescence “*in situ*” hybridization (FISH) analysis was performed using the following probes: dual color probe for chromosome 11 (LSI MLL dual color break apart rearrangement probe) and probe to* TP53* gene (LSI p53, spectrum orange). The probes were from Vysis, Abbott Laboratories, USA. Slide pretreatment, probe hybridization, posthybridization washing, and signal detection were done according to manufactured protocols. FISH analyses were done to confirm some chromosomal deletions as 11q23 and 17p and to characterize the breakpoint and the gene involved in the chromosomal abnormality. We used the samples of cytogenetic cultures.

### 2.3. Statistical Analysis

We studied the correlation between the karyotype and the clinical features. All the statistical analyses were done using the *χ* ^2^ test. A value of *P* < 0,05 was considered significant in all analyses. We studied statistically the following variables: age (≤18 years and >18 years), sex (male and female), MDS subtypes (RA/CR, RAEB, and RAEB-t), and risk groups with the frequency of normal versus abnormal karyotypes. We analyzed the association of frequency of abnormal karyotypes with the evolution of disease. Considering MDS subtypes, we analyzed the association of mortality with the frequency of abnormal karyotypes. For this, we classified our patients in two groups: RA/CR (initial stage of MDS) and RAEB or RAEB-t (later stages of MDS). We also studied the association between risk groups, according to the IPSS, and the frequency of abnormal karyotypes, evolution of disease, and mortality. In our study there were no patients classified as low risk IPSS subgroup. Although our patients were classified in three risk groups (intermediate-1, intermediate-2, and high risk), for the statistical analyses we considered two groups: group 1 and group 2. The elements in group 1 are the patients classified as intermediate-1 and the elements in group 2 were the patients classified as intermediate-2 or high risk. We also calculated the *P* value to study the evolution of disease and mortality comparing group 1 with group 2.

## 3. Results

### 3.1. Clonal Chromosomal Abnormalities in Patients with Hypocellular Primary MDS

**Figure 1:**
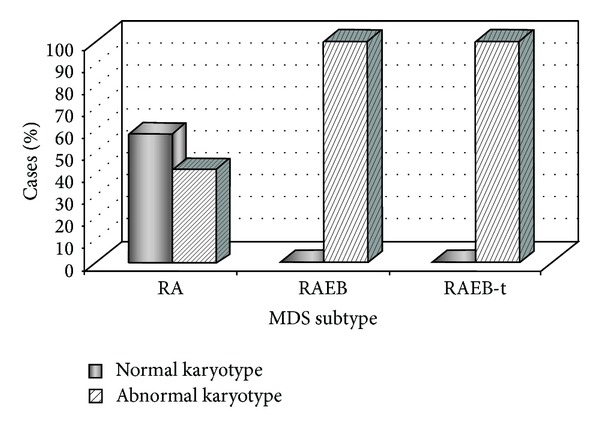
Frequency of normal versus abnormal chromosomal findings in subtypes of hypocellular primary MDS.

From a total of 482 patients, 105 patients showed hypocellular primary MDS, representing 21,8% of all cases. Among a total of 105 hypocellular primary MDS patients, clonal chromosomal abnormalities were detected in fifty-eight patients (55%). The distribution of abnormal karyotypes in each FAB subgroup was 42% in RA, 100% in RAEB, and 100% in RAEB-t (Figure 1). The frequency of abnormal karyotypes was significantly higher in later stages of disease (RAEB and RAEB-t) compared to the initial stage (RA) (*P* < 0,0001). Cytogenetic results showed that patients with RA presented normal karyotypes or single abnormalities as del(1q), del(3q), inv(3q), del(4q), del(5q), del(6q), del(7q), −7, del(9p), del(11q), del(12p), del(17p), hyperdiploid karyotype, biclonal chromosomal abnormality, and a marker chromosome. In the RAEB and RAEB-t group, single chromosomal abnormalities were observed such as −5, del(6q), +6, del(7q), −7, i(7q), t(7;9), +8, i(9q), del(11q), del(17p), del(20q), and complex karyotypes. As we can see in Table 1, there was no specific chromosomal abnormality associated with a subtype of hypocellular primary MDS. We analyzed 105 patients with hypocellular primary MDS. Among 39 pediatric patients with hypocellular primary MDS, 27 (69%) had abnormal karyotypes and among 66 adult patients, 31 (47%) showed abnormal karyotypes. The pediatric MDS has different frequencies of chromosomal abnormalities comparing to adult MDS. In pediatric patients, the chromosomal abnormalities that had higher frequencies were del(7q)/−7, del(11)(q23), and del(17p) and in adult patients were del(5q), +8, and del(17p). All cases of del(11)(q23) and del(17p) were confirmed by FISH analyses. These analyses showed the deletion of one allele of* MLL* gene in 11q23 and of* TP53* gene in 17p, confirming the results of conventional cytogenetic, the G-banding.

From 105 patients, 19 (18%) showed progression from MDS to AML. The median time for AML transformation was 2,5 months. The median and mean of overall survival in 105 patients were 35 and 52 months, respectively. The chromosomal abnormalities presented in the diagnosis of patients, who showed evolution of disease, included the numerical chromosomal abnormalities −7, +8, and the structural abnormalities del(6q), del(7q), i(7q), t(7;9), i(9q), del(11q), and complex karyotypes. During the evolution of the disease, we observed the gain of the chromosomal abnormalities del(7p), i(9q), dup(1q), del(11)(q23), and +8.

### 3.2. Correlation of Karyotypes and Clinical Features in Patients with Hypocellular Primary MDS

We analyzed different variables in patients with primary MDS as age, sex, MDS subtypes, and distribution of risk groups according to IPSS with the presence of normal versus abnormal karyotypes (Table 2). In our study, we observed some extremely significant results. One is that advanced stages (RAEB and RAEB-t) had a highly significant association with the frequency of abnormal karyotypes (*P* < 0,0002), evolution of disease (*P* < 0,0001), and mortality (*P* < 0,0005). Considering the patients according to group 1 (intermediate-1) and group 2 (intermediate-2 and high risk), we also showed that group 2 had a high association with respect to the frequency of abnormal karyotypes (*P* < 0,0001), evolution of disease (*P* < 0,0001), and mortality (*P* < 0,001). Our patients received different treatments: chemotherapy, supportive care, immunosuppressive therapy (ATG/cyclosporine), allogeneic hematopoietic stem cell transplantation, and others (thalidomide, lenalidomide). In our study, we verified that patients treated with allogeneic stem cell transplantation achieved a better treatment response.

## 4. Discussion

The hypocellular primary MDS is a rare neoplastic disease. In this study, we analyzed the chromosomal abnormalities of 105 patients with hypocellular primary MDS and the clinical features. In our study, the frequency of abnormal karyotypes was 55%. This result was similar to the studies of Yue and colleagues [6] and Huang and colleagues [12]. In the first study, it analyzed 163 patients and it found 47,5% of abnormal karyotypes [6]. In the second study, in a total of 33 patients, it found 42,2% of cases with abnormal karyotypes [12]. However, Marisavljević and colleagues showed a small frequency of cases with abnormal karyotypes (12,5%) [11]. We think this result may be associated with the low number of patients studied: 24 patients. Although the authors of these studies showed the frequency of abnormal karyotypes in hypocellular MDS, they did not discuss specific chromosomal abnormalities and their correlation with leukemic transformation.

The main chromosomal abnormalities found in our patients were del(5q), del(7q), −7, +8, del(11q), del(17p), and complex karyotypes. These chromosomal alterations are similar to those found in normo-/hypercellular. So, in primary MDS, independently of the cellularity in the bone marrow, cytogenetic pattern is characterized mainly by losses of partial or total chromosomes, suggesting that the main class of genes involved in the pathogenesis of MDS is the tumor suppressor genes. In the context of tumor suppressor genes, both alleles usually must be inactivated according to Knudson's two-hit hypothesis, wherein one allele is often deleted, and the other allele is inactivated either by deletion, mutation, or epigenetic modification. However, chromosome deletions, such as del(5q) in MDS, have introduced the concept of haploinsufficiency where there is a monoallelic inactivation, for example, the* RPS4* gene that is involved in the development of del(5q) MDS [19]. The most frequent chromosomal alteration found in our study was del(17p) associated with the deletion of one allele of the tumor suppressor gene* TP53*. The* TP53* gene has been described as “the guardian of the genome,” because of its role in maintaining the chromosomal stability. Deletion 17p has been reported in approximately 15% of the* novo* MDS cases. Alterations in this gene have been reported to be a later genetic event in the carcinogenesis model established in colorectal cancer and in chronic myeloid leukemia. However, in primary MDS alterations in* TP53*, like chromosomal deletions, may be detected in early stages. So, in those patients with a* TP53* alteration, a careful follow-up seems to be necessary because of the risk of early leukemic transformation. And a more intensive treatment may have to be considered for these patients [20–22]. In our study, most patients with del(17p) were at the early MDS stage, RA/RC. Most of these patients were pediatric and they were treated with hematopoietic stem cell transplantation. Silveira and colleagues suggested that* TP53* deletion in MDS represents a clinically relevant biomarker, which could be used to define* de novo* pediatric MDS [21].

Although the hypocellular MDS shows a low frequency of disease evolution [6, 11, 12], it is important to elucidate the chromosomal alterations associated with leukemic transformation, because the numerical and structural chromosomal abnormalities involved for the leukemic transformation in the cases of hypocellular primary MDS are still unknown. In our study, from 105 patients analyzed, 19 (18%) showed evolution of disease. The chromosomal abnormalities presented in the diagnosis of patients who showed evolution from MDS to AML included the numerical chromosomal abnormalities −7, +8 and the structural abnormalities del(6q), del(7q), i(7q), t(7;9), i(9q), del(11q), and complex karyotypes. During the evolution of the disease, we observed the gain of the chromosomal abnormalities del(7p), i(9q), dup(1q), del(11)(q23), and +8. Some of these chromosomal alterations, such as dup(1q), +8, and del(11)(q23), were previously described by our group as involved in the evolution from MDS to AML [10]. It is interesting to observe that important genes involved in the hematopoietic process, cell cycle control, and epigenetic control are in the regions of these chromosomal alterations. The members of* Hox* gene family,* HoxA9* and* HoxA10*, are localized in 7p15. These genes are expressed in hematopoietic precursors, with preferential expression in selfrenewing hematopoietic stem cells (HSC) and downregulation during terminal differentiation. The dysregulation of* Hox* genes is associated with a number of malignancies including the AML [23]. Deletions of 9p21 have been detected in various tumor types. The p15*INK4B* and p16*INK4A* genes are members of the cyclin-dependent kinase (CDK) inhibitor family, which control progression of the cell cycle from G1 to S phase. These genes are located in the 9p21 region. It also has been found that methylation in the promoter region of these genes is involved in evolution from MDS to AML [24]. Another chromosomal abnormality observed during leukemic transformation was the duplication of the long arm of chromosome 1. The dup(1q) has been associated with leukemic transformation in MDS as the unique cytogenetic event or associated with other chromosomal abnormalities [25]. In the long arm of chromosome 1, there are important genes that may be involved in the leukemic transformation. However, the* BCL9* gene, mapped in the 1q21, plays an important role in the* Wnt* signaling pathway and it is associated with tumor progression. This pathway is evolutionary conserved. At cellular level, this pathway regulates morphology, proliferation, and cell fate [26]. We also observed the del(11)(q23) involved in cases of evolution of disease. The* MLL* gene is mapped in the 11q23 region. The* MLL* gene is associated with various hematologic malignancies but is particularly common in infant [27]. Trisomy 8 was another chromosomal abnormality observed in the evolution of disease. Our group had already suggested that the gene probably involved in this leukemic transformation is the c-myc mapped in 8q24 [28]. It is interesting to observe that the hypocellular primary MDS has as chromosome alterations involved in leukemic evolution as hyper- and normocellular MDS. Our results suggest that probably the pathways of leukemic transformation may be the same.

We applied the IPSS in our patients with hypocellular MDS. According to the risk group stratification our patients were distributed in intermediate-1, intermediate-2, and high risk group. We did not have patients classified as low risk group, because in our sample all the patients had at least two cytopenias, receiving the classification of intermediate-1. This score system has been proved to be useful for predicting evolution of disease. When we considered the patients according to group 1 (intermediate-1) and group 2 (intermediate-2 and high risk), we showed that group 2 had a high association with respect to the frequency of abnormal karyotypes (*P* < 0,0001), evolution of disease (*P* < 0,0001), and mortality (*P* < 0,001). The IPSS is an important standard for assessing prognosis of primary MDS. And recently, this score system was revised and multiple statistically weighted clinical features were used to generate a prognostic categorization model. But bone marrow cytogenetics, marrow blasts percentage, and cytopenias remained the basis of the revised system [29].

Rare cytogenetic abnormalities, considered as intermediate group in the IPSS, as hyperdiploidy and cytogenetic biclonality, already described by our group, may be reported to help to elucidate its clinical implications in hypocellular primary MDS [30, 31]. In these studies we showed the importance of cytogenetic abnormality for the diagnosis of hypocellular primary MDS and to indicate the patients to stem cell transplantation. It is important to note that, in some cases, the hypocellular bone marrow makes the diagnosis between MDS and aplastic anemia a difficult process, and the cytogenetic, in these cases, is considered an important tool for diagnosis characterizing a clonal chromosomal abnormality and indicating the diagnosis of MDS [30, 32].

In our patients, the majority of mortality was not associated with evolution of disease, but with the cytopenias associated with infections, anemia, and hemorragie. So, in hypocellular primary MDS, it is important to analyze the frequency of transfusions and the life quality of the patients. Regarding this point, recently Tong and collaborators showed that patients with hypocellular MDS presented more frequently with thrombocytopenia, neutropenia, increased transfusion dependency and intermediate-2/high risk disease compared with patients with hyper-/normocellular MDS [33]. However, in our study, the most of patients were classified in intermediate-1; they were in the initial stage of MDS (RA/RC).

The rates of allogeneic stem cell transplantation (SCT) to treat MDS are continually increasing [34]. The allogeneic SCT is the only treatment modality that has been demonstrated to cure patients with MDS [35]. However, given the variety of therapeutic options in parallel to the heterogeneity of MDS, determining the indications for SCT in MDS is considered a difficult task [34]. Based on cytogenetic and clinical studies our patients were indicated for bone marrow transplantation at initial stages of MDS, where the cytogenetic abnormalities play an important role aiding to indicate and select these patients for this treatment, specially the pediatric patients. So, the cytogenetic gives more precision in deciding the treatment with bone marrow transplantation. It has been suggested that allogeneic HSCT offers optimal survival benefits when the procedure is performed before MDS patients progress to advanced disease stages [36]. Our study provides new information into the role of the chromosomal abnormalities in hypocellular primary MDS with important clinical implications. The cytogenetic analysis is an important laboratory tool for diagnosis, prognosis, in clinical decision-making and in follow-up for pediatric and adult patients with hypocellular primary MDS.

New cytogenetic methods like FISH, array-CGH (comparative genomic hybridization), SKY (spectral karyotype), and MCB (multicolor banding) are considered complementary analyses for conventional cytogenetic. The diagnostic workup for MDS now frequently includes FISH panels using multiple probes for most balanced chromosomal defects. Since FISH can be performed on interphase nuclei, these panels allow for target detection of specific chromosomal abnormalities even when metaphase cytogenetics is not possible because of no mitosis [37]. The molecular cytogenetic methods as MCB allowed the characterization and provided the ability to identify candidate genes involved in the leukemogenesis process in MDS [38].

As clonal chromosomal abnormalities were observed in about 50% of MDS patients, the necessity of new additional biomarkers that aid the diagnosis and prognosis for MDS is clear [39]. The new karyotyping and molecular tests, such as chromosomal microarray analysis, next generation sequencing (NGS), have increased the detection of genetic abnormalities in MDS and increased our understanding on the MDS biology. But these new genetic methods are being used mainly in basic research. Although new methods are potentially diagnostic tools, they still have not replaced the traditional laboratory techniques such as conventional cytogenetic and FISH analyses. Several studies point that cytogenetic analysis is still the gold standard genetic laboratory testing for diagnosis and prognosis in myelodysplastic syndrome [7, 15, 29]. Another important point is that chromosome banding remains the only low-cost genome screening technique, allowing the identification of balanced as well as unbalanced genomic rearrangements in single cells [40].

## 5. Conclusions

The remarkable progress in understanding the leukemogenesis was sustained by methodological developments in the cytogenetic field. In several studies, the cytogenetic turned out to be one of the most important prognostic parameters and it was incorporated into statistical models aiming a prognostic scoring system, like the International Prognostic Scoring System (IPSS) for myelodysplastic syndrome (MDS). Because MDS is a very heterogeneous disease, the diagnosis and the prognosis are generally considered a difficult clinical practice. In this study, we analyzed the chromosomal abnormalities of 105 patients with hypocellular primary MDS and the clinical features. The main chromosomal abnormalities found in our patients were del(5q), del(7q), −7, +8, del(11q), del(17p), and complex karyotypes. In our study, from 105 patients analyzed, 19 (18%) showed evolution of disease. The chromosomal abnormalities presented in the diagnosis of patients who showed evolution from MDS to AML included the numerical chromosomal abnormalities −7, +8 and the structural abnormalities del(6q), del(7q), i(7q), t(7;9), i(9q), del(11q), and complex karyotypes. During the evolution of the disease, we observed the gain of the chromosomal abnormalities del(7p), i(9q), dup(1q), del(11)(q23), and +8. It is interesting to observe that important genes involved in the hematopoietic process, cell cycle control, and epigenetic control are in the regions of these chromosomal alterations. Our study provides new information into the role of the chromosomal abnormalities in hypocellular primary MDS with important clinical implications. The cytogenetic analysis is an important laboratory tool for diagnosis, prognosis, in clinical decision-making and in follow-up for pediatric and adult patients with hypocellular primary MDS.

## Figures and Tables

**Table 1:** Cytogenetic analysis in 105 patients with hypocellular primary MDS.

Case	Age (years)	FAB	Karyotype	IPSS	Evolution from MDS to AML/time to AML transformation (months)
1	7	RA	46, XX[25]	Int-1	No
2	16	RA	46, XY[33]	Int-1	No
3	53	RA	46, XY[42]	Int-1	No
4	61	RA	46, XX[20]	Int-1	No
5	17	RA	46, XX[20]	Int-1	No
6	12	RA	46, XX[49]	Int-1	No
7	27	RA	46, XY, del(17)(p12)[5]/46, XY[23]	Int-1	No
8	26	RA	46, XX[30]	Int-1	No
9	22	RA	46, XY[25]	Int-1	No
10	7	RA	46, XX[31]	Int-1	No
11	22	RA	46, XX[22]	Int-1	No
12	32	RA	46, XY[28]	Int-1	No
13	9	RA	46, XX, del(12)(p12)[5]/46, XX[15]	Int-1	No
14	36	RA	46, XX[23]	Int-1	No
15	43	RA	46, XX[25]	Int-1	No
16	23	RA	46, XX[30]	Int-1	No
17	32	RA	46, XY, del(6)(q21)[3]/46, XY[21]	Int-1	No
18	31	RA	46, XY, del(17)(p12)[6]/46, XY[24]	Int-1	No
19	51	RA	46, XY, del(17)(p12)[8]/46, XY[20]	Int-1	No
20	14	RA	46, XY, del(17)(p12)[13]/46, XY[36]	Int-1	No
21	15	RA	46, XY, del(17)(p12)[4]/46, XY[18]	Int-1	No
22	37	RA	46, XX[30]	Int-1	No
23	16	RA	51, XX, +4, +6, +8, +14, +20[3]/46, XX[41]	Int-1	No
24	12	RA	46, XY, del(17)(p12)[4]/46, XY[17]	Int-1	No
25	16	RA	46, XX, inv(3)(q21q26)[5]/46, XX[15]	Int-1	No
26	84	RA	46, XY, del(17)(p12)[4]/46, XY[16]	Int-1	No
27	19	RA	46, XY[32]	Int-1	No
28	29	RA	46, XY, del(17)(p12)[6]/46, XY[19]	Int-1	No
29	62	RA	46, XX[29]	Int-1	No
30	18	RA	46, XY, del(1)(q32)[8]/46, XY[16]	Int-1	No
31	51	RA	46, XY, del(11)(q23)[8]/46, XY[17]	Int-1	No
32	30	RA	46, XX, del(17)(p12)[5]/46, XX[16]	Int-1	No
33	31	RA	46, XY, del(11)(q23)[4]/46, XY[17]	Int-1	No
34	13	RA	46, XY, del(7)(q22)[7]/46, XX[14]	Int-2	Yes/6
35	13	RA	46, XY, del(12)(p12)[4]/46, XY[36]	Int-1	No
36	49	RA	46, XX[20]	Int-1	No
37	9	RA	46, XY, del(3)(q23)[4]/46, XY[13]	Int-1	No
38	10	RA	46, XX[34]	Int-1	No
39	42	RA	46, XY, del(17)(p12)[6]/46, XY[16]	Int-1	No
40	32	RA	46, XY[27]	Int-1	No
41	48	RA	46, XY[20]	Int-1	No
42	41	RA	46, XX[20]	Int-1	No
43	56	RA	46, XX[22]	Int-1	No
44	34	RA	46, XY[22]	Int-1	No
45	10	RA	46, XY, del(17)(p12)[5]/46, XY[16]	Int-1	No
46	24	RA	46, XX, del(17)(p12)[3]/46, XX[20]	Int-1	No
47	19	RA	46, XY[30]	Int-1	No
48	29	RA	46, XX[34]	Int-1	No
49	11	RA	46, XY[20]	Int-1	No
50	4	RA	47, XY, +mar[3]/46, XY[25]	Int-1	No
51	38	RA	46, XX[20]	Int-1	No
52	26	RA	46, XX[42]	Int-1	No
53	27	RA	46, XX[20]	Int-1	No
54	7	RA	46, XY, del(12)(p12)[3]/46, XX[27]	Int-1	No
55	13	RA	46, XY, del(17)(p12)[9]/46, XY, del(17)(p12), del(12)(p13)[5]/46, XY, del(11)(q23)[3]/46, XY[34]	Int-1	No
56	45	RA	46, XX[20]	Int-1	No
57	18	RA	46, XX, del(9)(p21)[4]/46, XX[17]	Int-1	No
58	44	RA	46, XY[22]	Int-1	No
59	48	RA	46, XX, del(5)(q13q33)[7]/46, XX[13]	Int-1	No
60	32	RA	46, XY[30]	Int-1	No
61	72	RA	46, XX[21]	Int-1	No
62	56	RA	46, XX[30]	Int-1	No
63	42	RA	46, XY[32]	Int-1	No
64	7	RA	46, XY, del(11)(q23)[4]/46, XY[48]	Int-1	No
65	2	RA	46, XX, del(4)(q22)[4]/46, XX[28]	Int-1	No
66	14	RA	46, XY[50]	Int-1	No
67	61	RA	46, XX, del(5)(q13q33)[6]/46, XX[21]	Int-1	No
68	36	RA	46, XY[22]	Int-1	No
69	12	RA	46, XY[30]	Int-1	No
70	29	RA	46, XX[21]	Int-1	No
71	11	RA	46, XX[36]	Int-1	No
72	52	RA	46, XY[25]	Int-1	No
73	58	RA	46, XX, del(5)(q13q33)[16]/46, XX[7]	Int-1	No
74	36	RA	46, XY[28]	Int-1	No
75	12	RA	46, XY[30]	Int-1	No
76	49	RA	46, XY[27]	Int-1	No
77	10	RA	45, XY, −7[16]/46, XY[6]	Int-2	Yes/4
78	13	RA	45, XY, −7[8]/46, XY[12]	Int-2	No
79	37	RA	46, XX[24]	Int-1	No
80	10	RA	46, XX, del(6)(q24)[3]/46, XX[15]	Int-1	No
81	17	RA	46, XX[20]	Int-1	No
82	11	RAEB	46, XY, del(11)(q23)[7]/46, XY[13]	Int-2	Yes/4.5
83	6	RAEB	46, XX, i(9)(q10)[15]/46, XX[7]	Int-2	Yes/1
84	56	RAEB	46, XX, dup(1)(q12)[18]/45, XX, dup(1) (q12), del(3)(q23), −5[3]/46, XX[42]	Int-2	Yes/2
85	44	RAEB	46, XX, del(17)(p12)[8]/46, XX[14]	Int-2	No
86	63	RAEB	46, XX, del(17)(p12)[5]/46, XX[45]	Int-2	No
87	58	RAEB	46, XY, del(20)(q11)[29]/46, XY[2]	Int-2	No
88	17	RAEB	46, XY, del(11)(q23)[9]/46, XY[13]	Int-2	Yes/4
89	45	RAEB	46, XY, del(17)(p12)[8]/46, XY[14]	Int-2	No
90	41	RAEB	46, XY, del(11)(q23)[8]/46, XY[16]	Int-2	No
91	15	RAEB	46, XX, del(11)(q23)[8]/46, XX[16]	Int-2	Yes/3
92	42	RAEB	46, XX, del(5)(q21), del(8)(q22)[14]/46, XX, del(5)(q21), del(8)(q22), i(9)(q10)[9]	High	Yes/1
93	60	RAEB	47, XY, +8[32]/46, XY[23]	Int-2	Yes/2.5
94	58	RAEB	46, XX, t(7; 9)(q32; q34)[3]/46, XX[29]	High	Yes/3
95	19	RAEB	46, XX, i(7)(q10)[4]/46, XX[17]	High	Yes/1
96	61	RAEB	46, XX, del(6)(q21)[13]/46, XX[9]	Int-2	Yes/2
97	58	RAEB	45, XY, del(7)(q22)[16]/46, XY[5]	High	Yes/3
98	11	RAEB	45, XY, −7[25]/46, XY[3]	High	Yes/1
99	49	RAEB	47, XY, +6[8]/46, XX[20]	Int-2	No
100	55	RAEB	45, XX, −5[6]/46, XX[18]	Int-2	No
101	1	RAEB-t	45, XX, −7[24]/46, XX[2]	High	Yes/1
102	42	RAEB-t	46, XY, del(6)(q21)[5]/46, XY[18]	High	Yes/5
103	64	RAEB-t	46, XY, t(1; 6)(p23; p25)[16]/46, XY, t(1; 6) (p23; p25), del(7)(p25), i(9)(q10)[2]/46, XY[11]	High	Yes/1
104	57	RAEB-t	47, XY, +8[14]/46, XY[8]	High	Yes/2
105	7	RAEB-t	46, XY, del(11)(q23)[12]/46, XY[13]	High	Yes/3.5

**Table 2:** Correlation of karyotypes and the clinical features in patients with hypocellular primary MDS.

Patient's variables	Number of patients/frequency (%)	Karyotypes (%)		*P* value	Evolution from MDS to AML	*P* value	Mortality	*P* value
		Normal	Abnormal					
Age				*P* < 0,05		NS∗		*P* < 0,05
≤18 years (pediatric patients)	39 (37%)	12 (31%)	27 (69%)		39/9		39/12	
>18 years (adult patients)	66 (63%)	35 (53%)	31 (47%)		66/10		66/36	
Sex				NS∗		NS∗		NS∗
Male	56 (53%)	20 (36%)	36 (64%)		56/11		56/26	
Female	49 (47%)	27 (55%)	22 (45%)		49/8		49/22	
MDS subtypes/% of bone marrow blasts				*P* < 0,0002		*P* < 0,0001		*P* < 0,0005
Initial stage RA/<5%	81 (77%)	47 (58%)	34 (42%)		81/2		81/27	
Advantage stages RAEB and RAEB-t ≥5%	24 (23%)	0	24 (100%)		24/17		24/21	
IPSS				*P* < 0,0001		*P* < 0,0001		*P* < 0,001
Low	0 (0%)	—	—		—		—	
Int-1	78 (74%)	47 (60%)	31 (40%)		78/0		78/25	
Int-2	17 (16%)	0 (0%)	17 (100%)		17/9		17/13	
High	10 (10%)	0 (0%)	10 (100%)		10/10		10/10	

∗No significance.
